# Population Studies and Molecular Mechanisms of Human Radioadaptive Capabilities: Is It Time to Rethink Radiation Safety Standards?

**DOI:** 10.3390/ijms252413543

**Published:** 2024-12-18

**Authors:** Dmitry Vitalievich Sosin, Denis S. Baranovskii, Denis Nikolaevich Nechaev, Mariya Aleksandrovna Sosina, Alexander Vladimirovich Shaposhnikov, Georgy Aleksandrovich Trusov, Anastasia Germanovna Titova, Boris Fedorovich Krasnikov, Alexey Nikolaevich Lomov, Valentin Vladimirovich Makarov, Vladimir Sergeevich Yudin, Anton Arturovich Keskinov, Sergey Mihailovich Yudin, Ilya Dmitrievich Klabukov

**Affiliations:** 1 Federal State Budgetary Institution “Centre for Strategic Planning and Management of Biomedical Health Risks” of the Federal Medical Biological Agency, 119121 Moscow, Russiashaposhnikov@cspfmba.ru (A.V.S.); gtrusov@cspfmba.ru (G.A.T.); atitova@cspfmba.ru (A.G.T.); bkrasnikov@cspfmba.ru (B.F.K.);; 2 Department of Regenerative Medicine, National Medical Research Radiological Centre of the Ministry of Health of the Russian Federation, 249036 Obninsk, Russia; doc.baranovsky@gmail.com; 3 Research and Practical Clinical Center for Diagnostics and Telemedicine Technologies of the Moscow Health Care Department, 127051 Moscow, Russia; sosinama@zdrav.mos.ru

**Keywords:** radiation safety, radioresistance, hormesis, radioadaptive response, ionizing radiation, low-dose radiation, carcinogenesis

## Abstract

The evolution of man on Earth took place under conditions of constant exposure to background ionizing radiation (IR). From this point of view, it would be reasonable to hypothesize the existence of adaptive mechanisms that enable the human organism to safely interact with IR at levels approximating long-term natural background levels. In some situations, the successful operation of molecular mechanisms of protection against IR is observed at values significantly exceeding the natural background level, for example, in cancer cells. In 15–25% of cancer patients, cancer cells develop a phenotype that is resistant to high doses of IR. While further investigations are warranted, the current evidence suggests a strong probability of observing positive health effects, including an increased lifespan, a reduced cancer risk, and a decreased incidence of congenital pathologies, precisely at low doses of ionizing radiation. This review offers arguments primarily based on a phenomenological approach and critically reconsidering existing methodologies for assessing the biological risks of IR to human health. Currently, in the most economically developed countries, there are radiation safety rules that interpret low-dose radiation as a clearly negative environmental factor. Nowadays, this approach may pose significant challenges to the advancement of radiomedicine and introduce complexities in the regulation of IR sources. The review also examines molecular mechanisms that may play a key role in the formation of the positive effects of low-dose IR on human radioadaptive capabilities.

## 1. Introduction and Justification of the Theory of Radiation Hormesis

### 1.1. Historical Overview of the Modern Radiation Safety Model

The traditional approach to radiation protection and the assessment of the biological effects of radiation are based on two theories: the target theory and the linear non-threshold (LNT) model of radiation carcinogenesis. According to the target theory, which arose in 1920 and developed into its modern form by 1949 [1], the biological effects of radiation are a consequence of random and discrete absorption by sensitive components of cells (targets) of single elementary particles, photons or atomic nuclei, followed by the ionization of molecules and uncontrolled oxidation tissues through the free radical mechanism. With this model in mind, the LNT theory emerged in the 1940s, which implies that ionizing radiation is always harmful, no matter how small the dose. Most of the evidence supporting the LNT theory is based on extrapolations obtained from studies of people affected by the atomic bombings of Hiroshima and Nagasaki, as well as the Chernobyl disaster [2].

From its inception, the LNT model has been criticized, but has been accepted by the vast majority of regulatory authorities due to its simplicity, ease of use and technical inability to reliably determine the biological effects of low doses of radiation [3,4]. Note that the linear type of dependence for biological systems declared in LNT is extremely uncharacteristic. Biological systems contain a much larger number of components and types of interactions than technical systems using electricity or fluid mechanics [5]. However, creating a nonlinear model to describe the dose–effect relationship is extremely difficult. This is because conventional computational techniques (such as, for example, the analysis of normal vibrations, Fourier transforms, or the principle of superposition) do not work in nonlinear systems, due to the fact that the sum of the parts of such a system is not identical to the entire system as a whole [6]. Thus, at the current stage of development of biology, simpler phenomenological models can be used that do not necessarily require an understanding of the mechanisms of the system. A disadvantage of this approach is its lack of predictive power beyond the range of effects already studied. However, the current method of assessing health risks based on the LNT hypothesis not only introduces significant distortions into the understanding of the real state of affairs, but also leads to a distortion of the very principles of scientific analysis of health risks. For example, modern bioinformatics tools, such as adverse outcome pathway analysis (AOP), provide for the collection of data only on the negative effects of radiation, while positive effects are ignored [7]. In modern regulations, the use of the LNT model is still considered justified, primarily for safety reasons. However, to date, the LNT theory has not received significant experimental confirmation at low doses of IR and is largely based on extrapolation of reliable results of exposure to high doses of ionizing radiation. Proponents of the LNT model point to a number of significant circumstances that prevent its experimental confirmation when working with chronic low-dose radiation. Detecting an increase in the number of cancer patients in response to chronic low-dose radiation is extremely difficult given the high incidence of cancer in all human populations. This argument seems to be quite reasonable, so the selection of scientific data for this review relied primarily on studies with very large samples.

The challenges inherent in detecting and analyzing the effects of low-dose IR on human health yield two key implications. Firstly, an environmental factor whose influence on human health is indistinguishable from the background effects of other environmental factors may be reasonably excluded from consideration in regulatory health risk assessments.

Secondly, the difficulty of detecting negative effects of low doses of IR may be due to the fact that such effects of low doses of IR simply may not exist. A supposedly dangerous level of IR, which is significantly higher than modern background levels, has accompanied human evolution for millions of years [8]. 

Today, when nearly a hundred years have passed since the emergence of the LNT theory, two main contradictions of this theory are becoming more obvious and require attention from the scientific community, as follows:

1. The LNT theory essentially postulates the existence of an environmental factor, to which evolutionary adaptation for some reason did not occur or is impossible for humans (taking into account millions of years of evolution in the presence of the radiation factor).
2. The LNT concept implies that the currently known mechanisms of protection of biological macromolecules from damage, which are obviously ineffective at high doses of IR, are equally absolutely ineffective at low doses of IR (the condition of maintaining a linear dependence).

The following sections of this review provide examples of scientific studies that indicate the existence of a more complex system of interaction between the human body and ionizing radiation at low doses than was previously assumed. It is crucial to acknowledge that the type and rate of IR employed in each specific study can significantly influence the variability of observed biological effects, thereby impacting the reproducibility of results. Therefore, wherever feasible, equivalent doses have been reported to facilitate relatively accurate comparisons between studies.

### 1.2. Studies of LNT at Low Doses of Ionizing Radiation

Today, the multiple negative effects of high doses of ionizing radiation are beyond any doubt. At the same time, the number of publications reporting the negative effects of low doses of ionizing radiation and supporting the LNT concept is very small. Almost all studies of this nature are focused not on the identification of direct harm or a threat to health, but rather on the author’s interpretation of certain data as being potentially associated with a health risk. For example, an increase in markers of DNA double-strand breaks, γH2AX, has been interpreted as an increased risk factor [9], which is not obvious in the light of recent studies of human genomic DNA [9]. According to Tchurikov et al., DNA double-strand breaks appear to be one of the normal means of transcription regulation, and an increase in the number of DNA breaks in itself does not necessarily indicate negative consequences for human cells, but may be a marker of changes in the cellular transcriptional profile [10]. Similarly, the idea of an increase in reactive oxygen species in response to low-dose irradiation as a clearly negative phenomenon [11] also does not correspond to modern ideas about the molecular mechanisms of cell self-regulation [12]. In addition, a number of studies have shown a connection between an increase in the amount of reactive oxygen species and an increase in life expectancy [13].

Attempts to directly confirm the validity of LNT in low-dose radiation on large samples are difficult to call unambiguous. It has already been mentioned that mathematical methods do not yet allow for the full modeling of nonlinear biological processes. One of the consequences of this situation has been the appearance of a significant number of studies that attempt to use mathematical methods to prove the linearity of the dose–response relationship for low-dose IR, taking the LNT theory as an axiom. This approach undoubtedly leads to significant distortions in the assessment of biological processes. Let us consider several examples of such studies that can be used as confirmation of the LNT theory.

Two recent studies assessing various cancer risks among nuclear industry workers (INWORKS) reported finding statistically significant negative effects of low doses of IR on human health [14,15]. From a biological point of view, these studies contain several controversial approaches to data processing, which are highly likely to have led to significant distortions in the results obtained. First, the authors of the studies refused to use an external control group that was not exposed to radiation. That is, the study actually lacks a negative control, but only an internal positive control in the form of another irradiated group. Second, the authors extrapolate data based on the assumption of no threshold and consider only three hypothetical types of dose–effect dependencies: linear, linear–quadratic, and quadratic. In addition, all models initially assume the hypothesis that the minimum risk of cancer consequences will be observed at zero radiation levels. That is, the task of this type of research is formulated not from the position of searching for the type of dose–effect dependence, but from the position of searching for an answer to the question of whether it is possible to select such correction factors for the initial data set that will confirm the null hypothesis about the supposed type of dependence.

For the objectivity of the assessment, we will also pay attention to other aspects of planning experiments with the INWORKS group that could affect the final result. The authors of the studies themselves indicate that the estimates of radiation doses could contain errors leading to a bias in the results. In both studies, either approximate data from radiation monitoring results or readings from individual dosimeters were used to estimate the effective dose for workers in the nuclear industry. This methodology did not take into account the fact that workspaces at modern nuclear power plants are zoned by access level categories. The radiation background in the free access zone, where the vast majority of workers are located, and where wearing personal dosimeters is not mandatory, usually does not exceed or slightly exceeds the background values typical for the country on average (exposure estimates for these workers are based on stationary dosimeters measuring beta, gamma, and neutron radiation). While dosimeters are mandatory in controlled areas, not all risks are captured, as alpha radiation, which can significantly contribute to the effective dose, is typically not monitored in these areas. Other potential sources of bias include the use of death certificates to ascertain cause of death, particularly in Leuraud et al. [14], which analyzed leukemia subtypes. Additionally, the absence of smoking data in the leukemia study could introduce significant bias, given smoking’s role as a risk factor for myeloid leukemia. Consequently, the conclusions of both studies cannot be considered robust evidence supporting the LNT theory. In Leuraud et al. [14], the observed increase in risk falls within the margin of error for all leukemia types except chronic myeloid leukemia. In the study conducted by Richardson et al. [15], the authors’ conclusion that their results “suggest a linear increase in the relative rate of cancer with increasing exposure to radiation” can also be considered premature. The authors themselves note that “some evidence suggests a steeper slope for the dose–response association at lower doses than over the full dose”, a finding inconsistent with the LNT theory and known biological mechanisms of radiation protection.

Below are a few more examples. Over the past few years, quite a few studies have been devoted to analyzing publicly available data on mortality from various causes for a group of irradiated residents of Japan for the period 1958–2009 [16,17,18,19,20].

For the selected cohort of people, a retrospective assessment of the low-dose irradiation level was carried out, which critically depends on extrapolation from groups exposed to high doses and high-dose rates. As in most similar studies, a special mathematical apparatus based on the dose and dose-rate effectiveness factor (DDREF) was used for these purposes. An important component of DDREF is the factor determining the effect of dose extrapolation, the so-called low-dose extrapolation factor (LDEF). 

All of the aforementioned studies on the Japanese cohort of exposed individuals employed linear or linear-quadratic models to estimate LDEF. The effects of radiation exposure were assessed not through direct measurement but by selecting an appropriate excess relative risk (ERR) model based on mortality and morbidity data. This approach likely contributed to several artifacts in the final datasets. For example, in the work of Brenner and colleagues, who assumed a linear dose–response relationship [19], strong variability in ERR values was observed for different age groups, which, in fact, contradicts the LNT theory. In another study, Brenner and colleagues reported that, although the model generally correctly describes the dose–response relationship in the analyzed sample, a strong modification of the effect was observed depending on sex and age at exposure [20]. This result implies that the risk of radiation is not the same for all people, which is also not entirely consistent with the provisions of the LNT, since it raises questions about the existence of indirect effects of radiation. At the same time, the authors themselves write that the choice of the ERR estimation model and the observation period significantly affect the final results in the low-dose ranges. Furthermore, ERR estimates per Gy for all solid cancer mortality at low doses were sensitive to how background rates were modeled and adjusted and how the dose–response was controlled at higher doses. Therefore, it can be concluded that this study was not initially optimized for assessing risks at low doses of ionizing radiation. A similar study of kidney cancer incidence risks in the same cohort by Grant et al. [18] also revealed numerous inconsistencies when using linear and linear–quadratic dose–response models. For instance, when using a linear–quadratic model to estimate the ERR in men, a decreasing risk of kidney cancer incidence was found over a fairly wide range of radiation exposure from 0 to approximately 1.7 Gy. That is, mathematical modeling in such retrospective assessment of radiation data allows researchers to make many assumptions and obtain very different results depending on the chosen mathematical model. Thus, it should be recognized that, today, there are no statistically reliable experimental data on the harm to human health from ionizing radiation at an absorbed dose of ≤100 mGy.

### 1.3. Contradictions of Existing Models of Radiation Hormesis

**Figure 1:**
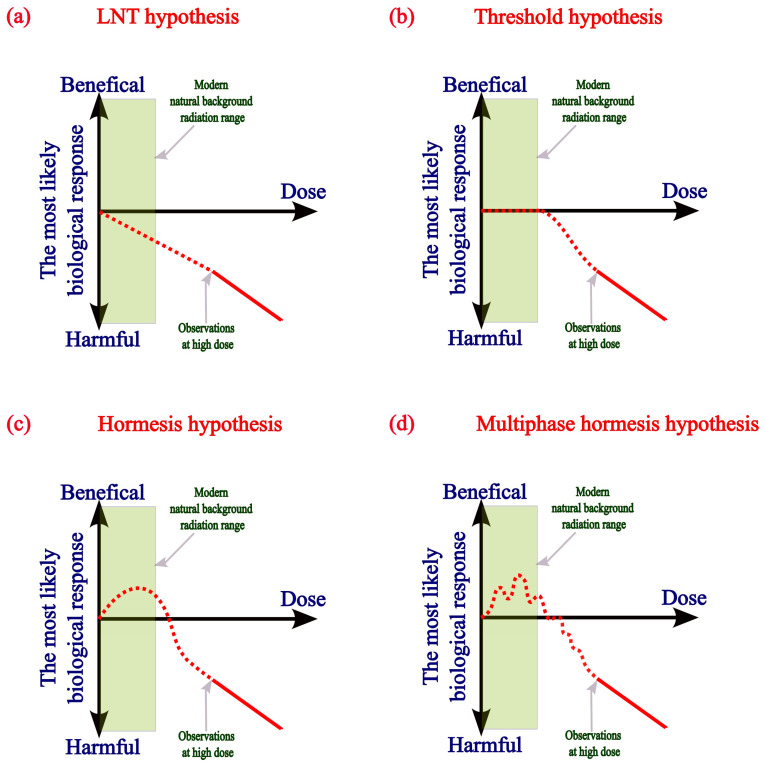
Graphical representation of the main hypotheses used to explain the biological effects of low-dose ionizing radiation. (**a**) Presented is a graphical diagram illustrating the linear increase in human health risks at any level of ionizing radiation (LNT hypothesis). (**b**) An illustration of the ionizing radiation threshold hypothesis, which states that negative effects on human health occur only above a certain dose rate and are then subject to linear dependence. (**c**) An illustration of the theory of radiation hormesis, according to which irradiation at dose loads close to the natural background has positive effects on human health. (**d**) The multiphase theory of hormesis explains the large scatter of data on biological effects observed by researchers when using similar dose loads, and also the observation of approximately the same biological effects at significantly different dose loads.

The target theory, which assumes the random and independent nature of events of damage to biological structures from IR, does not take into account that, with low-dose irradiation, some cells will not be affected by direct effects, and the main role in the cell response will be played by indirect effects, which are realized through intercellular signals and are not linear in nature. That is, with low-dose radiation, the expectation of a linear dose–effect relationship, which is declared both in the LNT theory and in the classical theory of radiation hormesis, is not justified. To complete the picture, it should be noted that existing models also do not explain the decrease in the harmful effects of radiation on the human body at high-dose rates (40–100 Gy/s) [21]. The existence of a system that can protect the human body with partial efficiency at high levels of IR is an indirect indication that, with a decrease in the dose load, the efficiency of such a system will be higher, but this issue requires further study. Thus, a theory that does not explain the observed effects, in accordance with the scientific model of cognition, requires clarification of the boundaries of its use, supplementation or complete revision. Several alternative hypotheses have been proposed by the scientific community to explain the effects of low-dose IR. The main current hypotheses used to explain the effects of low doses of radiation on the human body are presented in Figure 1.

To date, dozens of population studies have been conducted [22,23] that do not agree with the concept of LNT and suggest an alternative point of view regarding the existence of the phenomenon of hormesis when exposed to low doses of IR in humans. The essence of the concept of radiation hormesis is the existence of an adaptive two-phase response to IR, in which low doses lead to protective effects and improved performance of the body, while high doses of IR cause negative effects or the occurrence of pathologies [24,25]. Criticism of the hormesis theory is based mainly on the low reproducibility of experimental results with small samples; that is, large standard error values.

From the point of view of the nonlinear response to a stimulus characteristic of biological systems, we consider it reasonable to make additions to the existing model of radiation hormesis; namely, we assume that the absence of a linear relationship will be observed over the entire range of dose loads at which the biological mechanisms of adaptation to IR work effectively or with partial efficiency. That is, the multiphase nature of the dose–response curve will be observed both at the initial segment of the curve and in the region of suboptimal responses of the biological system to higher doses of IR (Figure 1d). In this case, data on a significant difference in biological effects with similar irradiation parameters become easily explained (vertical sections of the dependence curve), as well as data on the absence of significant differences in biological effects with different dose loads (horizontal sections of the dependence curve). Although today it is difficult to determine the exact parameters of radiation at which the stimulating effect of radiation changes to suppressive, there are good reasons to consider doses up to 100 mGy safe and even beneficial for the human body.

It is supposed that, at the initial stage of studying the effects of radiation, the use of the LNT hypothesis was justified, since it made it possible to save people’s lives through the most careful handling of radiation sources, but today this position is not objective and hinders the development of radiobiology. Strict adherence to LNT significantly diverts funding and expertise from potentially more fruitful areas of radiobiological research, such as understanding the effects of low doses of radiation on stimulating cell repair mechanisms, developing new radiopharmaceuticals, or advanced radiotherapy techniques [26]. Disentangling expenditures specifically attributable to the LNT model from general radiation safety practices, which would be necessary irrespective of the dose–response model employed, presents a significant challenge. However, in certain contexts, estimating the costs associated with low-dose IR source disposal is feasible. For example, according to the recommendations of the U.S. Environmental Protection Agency (EPA), all private homes should be regularly tested for radon levels [27]. Recognizing the hormesis theory will help determine the threshold values below which restrictions and regulations are not applied. In some states, including Mississippi, Louisiana, Arizona, and Florida, the average radon concentration in private residences does not exceed 1.9 pCi/L [28]. At the same time, there is reason to believe that statistically significant oncological risks arise at radon concentrations above 27 pCi/L [29].

Another example is the cost of decommissioning nuclear power plants. The International Atomic Energy Agency (IAEA) projects that approximately 200 nuclear reactors will initiate decommissioning by 2050. Decommissioning a nuclear power reactor, inclusive of waste management, typically incurs costs ranging from USD 500 million to USD 2 billion [30]. Gas-cooled, graphite-moderated reactors generally exhibit significantly higher decommissioning costs than pressurized water reactors (PWRs). Utilizing data from the U.S. Nuclear Regulatory Commission (NRC) reports on low-level radioactive waste (LLW) disposal costs associated with nuclear power plant decommissioning, and specifically referencing Table B-5 (“PWR Burial Costs at the Texas Site (2022 dollars)”) from NUREG-1307 [31], the estimated 2022 disposal cost for the safest class of radioactive waste (Class A LLW) from a reference PWR at the Texas disposal facility is USD 168,752,115. This figure encompasses taxes and fees but excludes transportation costs, as the disposal site falls within the generator’s compact region. Should low levels of IR be deemed safe for humans, a portion of the materials currently categorized as Class A LLW could potentially be directly recycled rather than disposed of via burial, given the absence of a minimum activity threshold for Class A waste in current U.S. regulations [32].

Attempts are still being made to explain the existence of hormetic effects by statistical biases in calculations, such as “survivor bias”, but none of the large-scale modern studies confirm this point of view [33]. That is, attempts are being made to deny this phenomenon on the basis that it does not fit into the obviously not entirely correct hypothesis. Two primary contradictions can currently be identified within the phenomenon of radiation hormesis. The first lies in the absence of clear criteria by which one can determine when irradiation parameters exceed the adaptive capacity of the biological system (i.e., which doses and irradiation regimens are threshold values for the transition from positive effects of IR to negative effects). To a large extent, this issue is resolved by defining the limit of low-dose IR guaranteed to be safe for humans and is discussed in more detail in the next section. The second contradiction of the classical hormesis hypothesis is associated with poor reproducibility of results under similar irradiation conditions. Apparently, this circumstance is associated both with the difficulties of modeling low-dose irradiation experiments and with the shortcomings of the hypothesis itself. In essence, the classical hormesis hypothesis suffers from the same flaw as the LNT hypothesis: it assumes a linear dose–response relationship throughout almost the entire downward portion of the dependence trajectory, which is not logical and does not correspond to a lot of experimental data [34]. The optimum performance of various cell protective systems falls within different ranges of low-dose IR. Therefore, it would be logical to assume that the entire dose–response curve would not be linear with low-dose IR; that is, in the range of effective operation of the cell’s protective systems, the dose–effect relationship will be multiphase in nature and contain areas of significant deviations from the linear relationship, as shown in Figure 1d. Thus, an important technical challenge remains the creation of model systems for studying chronic low-dose irradiation, which will identify and take into account all the factors influencing the susceptibility of a biological system to low-dose IR. In addition, differences in individual radiosensitivity of people to IR relate predominantly to dose values significantly exceeding the natural background, and have been reliably documented only for cohorts of people living in conditions of chronic exposure to damaging doses of ionizing radiation. Residents of the village of Dolon, Kazakhstan (mean annual effective dose: 2.37 mSv) exhibited an elevated frequency of chromosome aberrations (CAs) compared to a control group. However, G0 radiosensitivity, assessed by the level of CAs following in vitro irradiation of peripheral blood lymphocytes with a high dose (1 Gy), was lower than that of the control group, suggesting adaptation to chronic radiation exposure [35]. That is, the factor of individual radiosensitivity cannot be considered as a sufficient argument for applying the LNT hypothesis, since, over almost 80 years of research into the range of low doses of IR, assumptions about possible negative effects on human health have not been confirmed by scientific methods.

Thus, the main objectives of this review are as follows: firstly, to accelerate the recognition of the phenomenon of radiation hormesis as a basic scientific theory explaining the effects of ionizing radiation on living systems; and, secondly, to facilitate the removal of administrative barriers to the wider use of low-dose radiation sources in medical practice.

## 2. Published Evidence for the Theory of Radiation Hormesis

### 2.1. Population Studies of Hormesis

In the context of our review, the key characteristic of low-dose IR is the absence of statistically significant negative effects on human health. Although individual sensitivity to IR can vary greatly in the human population, in the vast majority of scientific studies, relatively safe doses of IR for humans are understood as parameters designated by the United Nations Scientific Committee on the Effects of Atomic Radiation (UNSCEAR), namely, an absorbed dose of ≤100 mGy at a dose rate of ≤0.1 mGy/min and low linear transfer energy [36]. Many population studies subsequently confirmed the validity of the parameters proposed by UNSCEAR, since modern population studies at radiation doses of up to 100 mSv (which for gamma radiation corresponds to an absorbed dose value of 100 mGy) do not reveal an increase in cancer incidence in humans [37,38]. Similar parameters for determining low-dose radiation have been proposed by other organizations and scientific communities influencing the formation of rules for handling sources of radiation, such as the International Commission on Radiological Protection (ICRP), the National Council on Radiation Protection and Measurement (NCRP), Multidisciplinary European Low Dose Initiative (MELODI) and Committee on the Biological Effects of Ionizing Radiation (BEIR) [39,40,41]. Thus, determining the limits of safe values for low-dose radiation is an important step towards legal recognition of the phenomenon of radiation hormesis.

This review uses the UNSCEAR interpretation of the concept of low-dose IR. However, some studies have reported the observation of hormetic effects at an absorbed radiation dose of about 500 mGy [42]. Most current studies draw conclusions about the effects of low-dose IR based on epidemiological studies of human and animal populations exposed to background radiation from nuclear power plants [43]. The results of these types of studies are difficult to interpret for a number of reasons, as follows: (i) a high level of sample heterogeneity, (ii) a small sample size or (iii) a very limited number of epidemiological markers [44,45]. PubMed contains more than 2000 scientific articles devoted to the study of the phenomenon of radiation hormesis in biological objects, but, in this section, information will be provided only on several of the largest studies performed on various cohorts of people.

David et al. [46] analyzed data on the effects of terrestrial and cosmic background radiation on the US population (more than 320 million people) in terms of correlations with human life expectancy and cancer mortality. Life expectancy (the most integrative indicator of population health) for people living in areas with relatively high versus low background radiation (≥1.8 mSv/year and ≤1 mSv/year, respectively; *p* < 0.005; 95% confidence interval (CI)) was approximately 2.5 years longer. The finding is largely due to the reduction in cancer mortality rates observed for several common types of cancer (lung, pancreatic and colon cancer in both sexes, and brain and bladder cancer in men only; *p* < 0.05; 95% CI). That is, exposure to high background radiation has clear hormetic effects, at least within the natural range of low radiation doses. Similar data were obtained by Indian researchers when studying the risks of developing congenital heart defects (CHDs) in newborns from areas with high levels of natural radiation (over 1.50 mGy/year) [47]. A total of 193,634 live newborns were included in the study. Multiple logistic regression analysis showed that the risk of congenital heart disease among newborns from the high-dose cohort of 1.51–3.0 mGy/year was significantly lower compared with the normal-dose cohort (odds ratio (OR)) = 0.72, 95% CI: 0.57–0.92), whereas it was similar in the very-high-dose cohorts of 3.01–6.00 mGy/year (OR = 0.55, 95% CI: 0.31–1.00) and ≥6.0 mGy/year (OR = 0.96, 95% CI: 0.50–1.85). The incidence of congenital heart disease did not show any tendency to increase with increasing radiation dose; on the contrary, there was a significant (*p* = 0.005) decrease in the prevalence of congenital heart disease in the group of newborns whose mothers lived in areas with high dose exposure (1.28%), compared with newborns whose mothers lived in areas with normal dose exposure (1.79%). In addition, the observed incidence of congenital heart disease in the high-dose cohort (1.49 per 1000 live births) was much lower than the previously reported average incidence of congenital heart disease in India (9 cases of congenital heart disease per 1000 live births [48]). It could be argued that perhaps the lower incidence of congenital heart disease is associated with a higher percentage of stillbirths in high-dose cohorts, but another large study of 141,540 newborns refutes this assumption [49]. The prevalence of stillbirths and major congenital anomalies among newborns in areas of India with high natural background IR is lower than in other areas of India. In this study, the rate of stillbirth was about 4 per 1000, which is approximately the level of most developed countries and is not typical for other regions of India [50]. The observed incidence of major congenital anomalies in regions with increased IR (about 1%) was also significantly lower than previously published data for other regions of India with lower IR rates (2.11–4.42%) [51,52,53,54].

Similar results were obtained in other observational studies. The largest epidemiological study of children born to parents of atomic bomb survivors in Japan (sample size: 71,603) did not find any significant effects of acute radiation exposure on the human population [55]. Parents in the study groups were stratified into the following four categories based on absorbed dose levels: <0.05, 0.05–0.49 Gy, 0.50–0.99 Gy, and ≥1.00 Gy. No statistically significant increase in the risk of congenital malformations or perinatal mortality was found in the groups as a whole. However, for the low dose, there was no increase in risk at all, and for the higher doses, only certain categories of malformations in some groups showed an increased risk. It should be noted that this study does not deal with statistical “survivorship bias”, since a significant proportion of people from the sample (2010 people) received high radiation doses above 0.5 Gy. Similarly, studies conducted in areas of the Chernobyl nuclear disaster did not show any increase in the prevalence of congenital malformations at birth. This study analyzed cohorts with an annual effective radiation doses below 100 μSv (from Belgium, Denmark and the Netherlands), as well as cohorts exposed to higher effective doses, such as 880–930 μSv (Ukraine) and 1900–2000 μSv (Belarus) [56]. Congenital heart defects after the Fukushima nuclear power plant accident also showed no increase in the number of congenital heart defects [57]. Another study consistent with hormesis theory was based on assessing the health consequences of the accidental contamination of load-bearing structures of 1700 apartments in Taiwan with cobalt-60 (T1/2 = 5.3 years) [58]. This case shows that chronic whole-body exposure to low-dose-rate radiation, even accumulated to a high annual dose, can be associated with demonstrable beneficial effects on human health. The cobalt-60-contaminated buildings housed approximately 10,000 people who unknowingly received an average radiation dose of 0.4 Sv over a period of 9–20 years. Despite the fact that many residents received quite high total doses of radiation—for example, 1100 people in 1983 received an average annual dose of 525 mSv—medical examinations did not reveal the presence of harmful radiation sickness syndromes, as was observed in survivors of the atomic bombs or in acutely exposed reactor workers after Chernobyl accident. No increases in the incidence of leukemia, congenital anomalies, adverse pregnancy outcomes or any other radiation-induced health effects were observed in European countries where whole-body doses received during the first year after the Chernobyl accident ranged from 0.05 to 0.5 mSv [59]. One group of specialists, studying residents of the Ming-Sheng Villa, a highly radioactive building, found that the frequency of micronuclei formation in the lymphocytes of exposed people (mean annual dose 26 mSv) was higher than in the control group, and that the ratio of lymphocytes in the blood of another group of residents (mean annual dose 24.8 mSv) differed from the ratio of lymphocytes in the control group [60,61]. In general, LNT proponents tend to interpret the molecular changes found with low doses of ionizing radiation (increased levels of inflammatory cytokines, decreased mast cell migration rates, increased frequency of micronuclei, decreased antioxidants and increased mitochondrial DNA 4977 bp deletion) as support for the LNT theory, without reversing attention to the absence of negative phenotypic manifestations at the organismal level [11,62,63]. A more cautious interpretation of these results is that low-dose, low-dose-rate gamma radiation from any radiation source causes cellular changes, but there is no indication that these changes had any adverse health effects. The general finding of the Atomic Energy Council of Taiwan (AEC) is that groups of individuals exposed to higher radiation doses had lower rates of chromosomal aberrations [64,65,66,67,68]. Summarizing the results of the studies by Chen et al., a conclusion can be made that the group of people exposed to chronic radiation did not experience higher cancer mortality [58], as predicted by the LNT theory. In contrast, the incidence of cancer deaths in this population was significantly reduced, with only 7 cancer deaths per 200,000 person-years compared with the Taiwanese average of 116, i.e., only 3% of the incidence of spontaneous cancer deaths in the Taiwanese population in general. In addition, the incidence of congenital malformations decreased to approximately 7% of the total incidence. If we exclude from the analysis 2000 students who were exposed to radiation only during classes in a contaminated building, then the number of cases of cancer in the remaining cohort of exposed people is reduced to 2.7% of the average, and the number of birth defects to 6.5% [58]. These data are in good agreement with the results of studying peripheral blood lymphocytes taken from residents of Dolon (a radiocontaminated village in the area of the Semipalatinsk test site, Russia). In this study, lymphocytes were irradiated in vitro with a dose of 1 Gy. It was found that the lymphocytes of Dolon residents were much more resistant to radiation and contained fewer chromosomal aberrations compared to the control group, which included residents from a similar-sized village in a neighboring region that was not part of a zone of increased radiation danger. That is, the adaptation of permanent residents of Dolon to high levels of background radiation is clearly manifested at the molecular level and their cells are better protected from damage to genomic DNA [33].

It should be noted that this review did not include population-based studies that examined the correlation between CT and cancer risk. Indeed, more than half of such studies found a statistically significant association between CT (with an IR dose of up to 100 mSv) and an increased risk of cancer. Comparison of cohorts of people who underwent CT and those who did not undergo this procedure does not seem correct as a tool for studying the oncogenic effects of IR, since the sampling is not homogeneous. A direct cause-and-effect relationship between a weakened immune system and an increased cancer risk is a generally accepted concept in modern science. At the same time, it is people with significant health problems who are usually referred for CT. That is, a small increase in cancer risks in the cohort of people who underwent CT most likely reflects the peculiarities of the immunological status within this cohort.

In 2006, the US National Academy of Sciences (NAS) attempted to rehabilitate the LNT theory in the BEIR VII report. The report’s conclusions are based on an investigation of the lifespan of atomic bomb survivors. Unfortunately, the researchers in this report did not take into account residual radiation, which led to underestimation of doses and erroneous selection of the control group. Residual radiation mainly consisted of radioactive fallout that fell to the ground along with the so-called black rain (Fallout Rain). According to Sutou’s estimations, residual radiation could have increased the total absorbed dose received after the nuclear detonation by a range of 0 to 750 mGy [69]. The areas affected by the black rain were extensive. Not only the survivors of the atomic bomb, but also people outside the city who were included in the control group should have been exposed to residual radiation to a greater or lesser extent. The study revealed no significant health risks associated with fallout exposure for either all-cause mortality or cancer mortality in Hiroshima. In Nagasaki, a weak correlation with all-cause mortality (ERR = 0.08) was observed between 1950 and 2005, but no significant increases in cancer mortality or incidence were detected [70]. Moreover, according to Sutou’s calculations, low doses of radiation prolonged the lives of atomic bomb survivors. Apparently, this is due to the fact that the approach proposed in BEIR VII takes into account only the suppression of DNA repair by high doses of radiation, but absolutely does not take into account the adaptive reactions of the body in response to low doses of radiation.

The absorbed dose of natural radiation of different populations is significantly influenced by altitude. The dose rate of cosmic radiation doubles every 1000–1500 m. In Europe, the annual effective dose values from cosmic radiation at ground level show a range from 301 to 3955 μSv depending on altitude and location. Maximum values (up to 3955 μSv) are recorded in high mountain areas such as the Alps and eastern Turkey (with altitudes above 3000 m). In 92% of cases, the population is exposed to doses below 400 μSv (altitude up to 700 m above sea level) [71]. A publication by Merrill and Frutos analyzed cancer registries covering 607 US counties [72]. This work showed that higher altitude as well as increased exposure to sunlight were associated with lower incidences of Hodgkin’s lymphoma and non-Hodgkin’s lymphoma (NHL), and the inverse association between sunlight and NHL was stronger with increasing altitude [72].

The biological effects of low doses of radiation may not be limited to reducing the risk of cancer or congenital pathologies. This is indirectly evidenced by a study by Xu et al., which showed a clear relationship between the altitude at which a person lives and the risk of developing diabetes mellitus or impaired glucose tolerance [73]. For example, compared with those living at altitudes < 3500 m, the risk of diabetes was found to be reduced by 65% for those living at altitudes of 3500–3999 m (*p* = 0.034) and by 89% for those lives at altitudes ≥ 4000 m (*p* = 0.015). It is likely that the discovered correlation may be associated with any mechanisms of adaptation to high altitudes, including, possibly, the phenomenon of radiation hormesis, which requires further study.

### 2.2. Molecular Mechanisms of Hormesis

In the late 1950s, radiation mutagenesis was considered the main negative consequence of ionizing radiation. Although more recent studies have not found an increased mutation rate in survivors of the nuclear bombings of Hiroshima and Nagasaki [74], most radiobiologists agree that DNA damage is a useful marker for determining the dominant type of response of a biological system to ionizing radiation [75]. Up to certain IR values, eukaryotic cells react predominantly at the level of changes in signaling pathways that do not affect the DNA repair system [76,77]. Apparently, the phenomenon of radiation hormesis is observed precisely at this level of dose loads. Consequently, indicators such as the total absorbed dose and dose rate (and, in general, the temporal patterns of radiation exposure) fade into the background during low-dose irradiation as long as the probability of DNA damage remains low. That is, one of the main drawbacks of the theory of radiation hormesis (namely, the low reproducibility of experiments) is largely associated with insufficient attention when planning experiments to such factors as the distribution of radiation sources and the structure and size of biological targets [34].

As soon as dose loads reach values at which the probability of damage to genomic DNA in exposed cells approaches one, the cellular response to radiation changes significantly [24,78,79]. It is important to understand that, although DNA damage itself may be random and independent (as described by target theory), the cellular response will not develop in a random or independent manner. This is due to the fact that, as a result of the effect of IR on biological tissues, not only does the direct ionization of biologically significant molecules occur, but also the launch of cascades of signaling processes (including in cells that have not been directly exposed to irradiation) [80,81]. Given that the signaling pathways involved in the response of biological systems to low-dose IR should have a low threshold for induction, the effects on the biological system may be difficult to predict.

In this review, we, like other researchers, consider the criteria of genomic DNA damage as a convenient indicator for determining the type of cellular responses that will be radically different from those during low-dose and high-dose IR [74]. Thus, the cell’s response to high or low levels of IR is determined not by the fact of damage to DNA or other molecules, but by the integrative cellular response. This kind of response includes many processes with nonlinear dynamics, for example, the launch of an inflammatory response when cells are damaged under the influence of DAMPs (damage-associated molecular patterns) molecules and many signaling pathways in response to DNA damage—DDR (DNA damage response) [82,83]. Moreover, it is necessary to take into account that the level of the cellular response of the human body to radiation will be largely determined by individual factors, such as, for example, the current level of physiological stress [84,85].

The use of the DNA integrity criterion as an indicator of low-dose radiation is also confirmed in population studies. For example, no significant effect of high background radiation in Kerala, India, on telomere length in newborns was found [86]. Another study showed that DNA damage caused by low doses of radiation is much less than damage caused by oxidative processes of normal metabolism [41,87]. Thus, it is the induction of signaling pathways that is one of the key features of the effects of low doses of radiation [4,88]. This fact is usually interpreted from the point of view of optimizing the body’s response to stress [89]. Indeed, many well-studied effects of low doses of IR at the cellular and molecular levels can be interpreted as adaptation to non-specific stress: increased DNA repair, the production of stress proteins, decreased reactive oxygen species (ROS), the release of growth factors, the activation of membrane receptors, compensatory cell proliferation, the removal of aberrant cells and the stimulation of the immune system [37,41,90,91,92]. Apparently, repair induced by low-dose irradiation can occur both through classical mechanisms and due to the formation of specific centers for DNA damage repair, which arise as preferred sites of repair. Such centers, commonly referred to as radiation-induced foci, are characterized by the local recruitment of p53-binding protein and other DNA damage-sensing proteins [93]. Several human studies have provided evidence that low-dose irradiation can lead to the activation of endogenous antioxidant defense components in various tissues, such as superoxide dismutase, catalase, glutathione, glutathione reductase and glutathione peroxidase [94,95]. However, most of the processes induced by low doses cannot be called specific. For example, the induction of Hsp70 and Hsp25 mRNA expression is a universal response of mammalian cells to most stress factors, including low-dose radiation [96,97].

It should be noted that many scientists in their studies, when studying the effects of low-dose IR, focus their attention on analyzing the activity of already known genes and signaling pathways (previously identified during acute irradiation with high doses of IR). This approach is largely a consequence of the dominance of the LNT paradigm in the scientific information space. However, the widespread use of transcriptomic and proteomic approaches is gradually shifting the emphasis to the discovery of new mechanisms of radioadaptation. There are also many nuances along this path. The effects of low-dose radiation, which are typically found with short-term and chronic exposure to human cells, differ significantly. For example, low-dose short-term irradiation of human whole blood in model experiments suggests that changes in the transcriptional profile predominantly affect genes involved in antigen processing and presentation [98,99]. However, transcriptomic analysis using the blood of people constantly living in conditions of high background radiation gives different results, and, in most cases, it is impossible to determine unambiguously whether changes in the expression level of a particular gene are associated with adaptation to chronic exposure or to other environmental factors [100,101]. In people living in areas of high natural background IR, researchers observe an increase in the expression of genes responsible for DNA repair, RNA processing, chromatin modification and cytoskeletal organization [100,101]. As mentioned above, proteins associated with DNA repair and maintaining chromatin stability play a far from leading role during chronic low-dose radiation (in this case we are talking about dose loads from 1 to ≥45 mGy/year). Analysis of protein–protein interactions revealed several proteins that apparently play an integrative role in adaptation to radiation stress: the YWHAZ protein, which is involved in the regulation of anti-apoptotic processes, the cytoskeletal protein ACTB and the molecular chaperone HSPA8. It should be noted that in all samples analyzed using liquid chromatography with tandem mass spectrometry and protein labeling using the iTRAQ (isobaric tags for relative and absolute quantification) method, no radical changes in protein abundance were observed between the control and experimental groups [100]. Nishad et al. showed that only a few of the 4166 unique proteins identified showed a greater than twofold increase or decrease in relative abundance in one or more experimental cohorts [100]. For example, in a cohort of people living under conditions of maximum dose rate (≥14.01 mGy/year), the greatest increase in the abundances of the proteins WNT10A, BLM and SRCN1, and a decrease in the abundances of RRP5, TBX2 and NF2IP proteins, were detected. It should be noted here that none of the listed proteins belong to the DNA repair system. In general, in comparison with acute irradiation, there are no sharp jumps in the profile of gene activity, which is one of the markers of the normal functioning of the human body as a single system. Similar results were demonstrated in a study by Jain et al. [101]. Despite the authors’ statement about the importance of changes in the activity of genes involved in DNA repair, none of them showed a change in activity of more than 1.3 times. At the same time, the overwhelming number of genes (for which a more than 1.3-fold change in activity was shown) encode transcription regulators and proteins involved in translation initiation, RNA processing, or protein degradation. Part of the important role of the protein degradation mechanism in radioresistance is suggested by recent experiments in which radioresistant cancer cells lost their resistance to ionizing radiation after the suppression of the protein SUMOylation system [102]. Currently, the role of SUMOylation in modulating the radioresistance of cancer cells is attributed to SUMO-specific proteases (SENPs) involved in the maturation of SUMO proteins [103], as well as SUMO-activating enzyme subunit 1 (SAE1) [104]. In particular, their role in DNA repair, p53 activity, apoptosis, proliferation, cell cycle and hypoxia is indicated [105].

It is impossible to claim that the identified differentially expressed genes are necessarily involved in the mechanism of radiation hormesis or play a key role in it. Such assumptions can only be verified by long-term direct experiments. A similar situation is observed with the discovery of polymorphisms in DNA repair genes in people exposed to chronic low-dose radiation [106], because proof of functionality of each of the detected polymorphisms is required.

One of the main features of low-dose IR is the presence of various non-target effects; that is, those that are found not only in those parts of the body that were directly irradiated, but can be found in neighboring cells and in the descendants of the irradiated organism. The main off-target effects include the activation of biochemical signaling pathways associated with the bystander effect, resistance to genomic instability and the induction of delayed lethal mutations [25,107,108,109]. Although the abscopal effect has been known for more than 50 years, currently, only a few mechanisms are known that ensure its operation at the molecular level. The phenomenon of abscopy is believed to be of immune origin and indicates that local radiotherapy (including low-dose irradiation) can cause systemic effects at the level of the whole body [110].

In 2011, Chen et al. demonstrated a new phenomenon known as the “radiation-induced rescue effect” (RIRE), whereby bystander cells rescued irradiated cells through cell–cell feedback, reducing cytotoxicity and genotoxicity caused by ionizing radiation. In experiments performed, cancer cells (HeLa) irradiated with α-particles were rescued by non-irradiated primary human lung fibroblasts (NHLF) in two-cell co-culture systems after 24-hour irradiation at doses of 20 or 40 cGy [111]. This finding raised concerns that the effectiveness of cancer treatment using ionizing radiation is compromised by a rescue effect from unirradiated normal cells, as RIRE has demonstrated a protective bystander effect [111,112,113]. Later, it was demonstrated that feedback from observer cells during irradiation can be dual in nature. The effect of weakening the harmful effects on cells on target cells when receiving feedback signals from observer cells is called the RIRE effect of the first type [111,112]. The second type of RIRE effect, on the contrary, exacerbates harmful effects in target cells when receiving feedback signals from bystander cells [112,114,115]. The manifestation of bystander effects, whether protective or deleterious, is contingent upon a complex interplay of factors. These include, but are not limited to, the phenotype and inherent characteristics of bystander cells, types of bystander factors, the types and quality of radiation, the biological endpoint and radiation dose [112,113].

According to one theory, mitochondria play a key role in signal transduction during low-dose IR. ROS generated by the IR or mitochondria at low, physiologically acceptable levels perform signaling and stimulating functions [12,75,116]. In normal cells, exposure to IR can indeed stimulate oxidative phosphorylation in mitochondria and, consequently, ATP synthesis [116,117,118,119,120]. At the same time, an increase in the energy actually available to the cell can trigger subsequent signaling cascades. This process is highly dependent on the oxidative and metabolic status of the cells (total number and mass of mitochondria, oxidative stress) and thus likely affects normal and cancer cells differently. Cancer cells are known to exhibit increased levels of oxidative stress and ROS production. One of the fastest cellular responses to oxidative stress is the direct modification of enzymes, increasing levels of the antioxidant glutathione or inactivating glutathione-dependent protective enzymes [121]. A few hours later, a hormetic response is observed at the transcriptional level through the up-regulation of Nrf2-mediated expression of enzymes involved in glutathione synthesis. It should be recognized that mitochondria can indeed influence many processes, the activation of which is observed during low-dose IR. For example, immunogenic antitumor responses such as the activation of macrophages and natural killer (NK) cells are regulated by mitochondrial activity [122]. However, damage to mitochondria by ROS can hardly be considered a specific trigger for triggering an adaptive response to low-dose IR, since radiation is far from the only cause of the appearance of excess amounts of ROS in the cell. In addition, it was already mentioned that in whole-transcriptome studies, there is no strong induction of genes encoding antioxidant proteins such as GPx, Trx, CAT and SOD2 (Mn-SOD). The transcriptional activity of such genes is regulated by the ROS-dependent transcription factor Nrf2 [123]. That is, the phenomenon of hormesis is much more complex, including many regulatory mechanisms, including at the epigenetic level in the form of alternative DNA methylation and induction of various non-coding RNAs [124]. Moreover, the induction of the immune system by long non-coding RNAs during low-dose IR can occur in an organ-specific manner [125]. Official recognition of the phenomenon of radiation hormesis will remove the “heretical” status of this topic and will help more researchers join the study of the effects of low-dose IR on living systems.

Numerous petitions with requests to revise radiation safety standards have not yet led to changes in regulatory documents; for example, NRC rejected three petitions on 17 August 2021 (PRM-20-28, PRM-20-29, PRM-20-30) on the revision of radiation safety standards taking into account radiation hormesis (document number 2021-17475). In May 2017, the Director of the National Council on Radiation Protection and Measurements, Dr. John D. Boice Jr., was forced to publish a clarification article emphasizing that the LNT concept remains a reasonable basis for approaching radiation safety standards, although it cannot be scientifically supported by radiobiological or epidemiological data [33].

## 3. Future Research

On the one hand, the modern scientific paradigm does not deny the possible presence of beneficial effects of low-dose IR, and on the other hand, it does not confirm their presence; nevertheless, in practice, we can see numerous examples of long-term use of this approach in medicine. X-rays and radium sources were widely used in the first half of the 20th century to treat a number of inflammatory diseases: arthritis, pneumonia, and much more [126]. Radiation therapy is currently used in different modes to treat cancer and prevent metastases [127]. Low-dose radiation is gradually returning to medicine and is showing its first successes. In the USA, a hospice patient with severe Alzheimer’s disease (and her husband with Parkinson’s disease) was successfully treated using CT scans of the brain [128]. Promising data have also been obtained on using chest x-rays to reverse the negative effects of acute respiratory distress syndrome in patients with COVID-19 [129,130,131,132]. Unfortunately, due to the nonlinear response to low-dose IR, health risk estimates remain difficult to predict and require detailed context-sensitive analysis [133,134].

Today, many scientists believe that a dose of 100 mGy can be considered safe for humans. However, to determine the biological effects of low-dose IR at higher dose values, it would be rational to conduct a more careful and targeted revision of the criteria for assessing the risk to human health, from the concept of “dose” to the concept of “response”. The cellular response to radiation today is the most predictive sign for determining exactly how the human body will react to the received dose of radiation. These reactions can be beneficial or harmful depending on many factors, some of which have already been studied. This means that promising models for assessing the risks of radiation injury must somehow take into account the factor of individual radiosensitivity. One of the most promising directions for determining the level of IR that is safe for humans is the analysis of non-coding RNAs [125]. Although most microRNAs alternatively expressed during low-dose IR are not clearly associated with radioprotective mechanisms, they are involved in the regulation of more than 60% of all human genes [135] and allow for a comprehensive assessment of the state of the human body. 

We believe it is important to emphasize that the purpose of this review is not to advocate the abolition of radiation safety standards and regulations. Clearly, monitoring potential radiation exposure of workers at nuclear power plants and in other relevant settings remains important. Rather, this review aims to initiate a discussion on the potential optimization of dosimetry and shielding techniques to achieve a balance between safety and cost-effectiveness based on the principles of radiation hormesis. For example, in the United Kingdom, the annual radiation exposure limit for workers in the nuclear industry is set at 20 mSv/year, but the actual average annual dose for this occupational group is 0.18 mSv/year [136]. This actual average is significantly lower than the standards set for other segments of the population (1 mSv/year) and is also substantially lower than the average annual dose received by individual occupational workers in the United States (6.2 mSv/year). Such discrepancies illustrate the need and highlight the opportunity to re-evaluate the current paradigm in which radiation doses as low as 100 mSv are considered dangerous while doses up to 50,000 mSv are used as routine therapeutic doses in breast cancer treatment [137].

## 4. Conclusions

The development of new approaches for the personalized assessment of health risks associated with low-dose IR remains one of the most controversial issues in the field. A successful solution to this problem may enable the wider application of the beneficial effects of IR in medical practice. The scientific community is practically on the threshold of possible recognition of the fact that there is currently no scientifically substantiated evidence calling into question the existence of the phenomenon of radiation hormesis. The lack of a detailed understanding of the mechanisms underlying a particular natural phenomenon is not yet a basis for limiting the application of this phenomenon to various aspects of human life. Well-known examples of such phenomena are superconductivity and gravity. Since the time of Paracelsus, medical science has fully come to understand that the therapeutic effect of any drug or exposure is maintained within a fairly narrow range. At present, there are good reasons to use this principle when working with ionizing radiation. It would be rational, given our current knowledge of radiation hormesis, to try to use it as a starting point for simplifying the regulatory framework and administrative systems of radiation control.
